# A novel automated label data extraction and data base generation system from herbarium specimen images using OCR and NER

**DOI:** 10.1038/s41598-023-50179-0

**Published:** 2024-01-02

**Authors:** Atsuko Takano, Theodor C. H. Cole, Hajime Konagai

**Affiliations:** 1 Institute of Natural Science and Environment, University of Hyogo/The Museum of Nature and Human Activities, Hyogo, 6 Chome, Yayoigaoka, Sanda, Hyogo 669-1546 Japan; 2 Institute of Biology, Dahlem Center of Plant Sciences, Freie Universität Berlin, Altensteinstrasse 6, 14195 Berlin, Germany; 3 Functions Tales, Shimogamo-Honmachi 19-1-101, Sakyo-ku, Kyoto, 606-0862 Japan

**Keywords:** Classification and taxonomy, Data acquisition, Machine learning, Software, Computational biology and bioinformatics

## Abstract

Digital extraction of label data from natural history specimens along with more efficient procedures of data entry and processing is essential for improving documentation and global information availability. Herbaria have made great advances in this direction lately. In this study, using optical character recognition (OCR) and named entity recognition (NER) techniques, we have been able to make further advancements towards fully automatic extraction of label data from herbarium specimen images. This system can be developed and run on a consumer grade desktop computer with standard specifications, and can also be applied to extracting label data from diverse kinds of natural history specimens, such as those in entomological collections. This system can facilitate the digitization and publication of natural history museum specimens around the world.

## Introduction

Natural history collections throughout the world hold some three billion specimens of preserved animals, plants, fungi, etc.^1^, but few have been digitally mobilized. The Global Biodiversity Information Facility (GBIF) now hosts 2.3 billion data items related to biodiversity (accessed 2023/04/13), but only 218 million of those are specimen based. Specimens of natural history collections are preserved actual organisms linked with manifold information on attached specimen labels: name of organism, collection site, collection date, collector’s name, and sometimes information on environment or habitat. They are primary evidence of the distribution and life history of an organism, providing insights into the history and evolution of the natural world. Therefore, increasing the distribution and availability of specimen information through digital imaging of natural history collections and the publication of specimen image archives on the web will facilitate future biodiversity research^2–4^.

To improve the availability and access to information on natural history specimens, it is necessary to promote the digitization of specimen information as metadata. Specimens in natural history museums and herbaria are increasingly being digitized all around the world^5–9^, and a simple, budget-friendly and efficient way of scanning specimens has been developed^10,11^. However, until now, data entry has been done manually by professional taxonomists, part-time workers or volunteers who are (more or less) familiar with the names of organisms, places, and collectors. For example, at the Naturalis Biodiversity Center in the Netherlands label data was entered manually by diverse collaborators from inhouse and abroad^12^. Such operations naturally require time and an adequate budget, of course. For herbarium specimens, OCR (optical character recognition: a technique that converts images of text into machine-readable text format) of label data has previously been applied to convert the label information from a specimen’s digital image to readable and electronically extractable text^13–18^. Semi-automatic extraction systems for specimen information such as HERBIS^19^ and SALIX^20^ have been developed. However, specimen labels contain diverse information such as plant names, collection sites, names of collectors, and collection dates, and plain OCR text, if simply extracted, would be a chaotic mix of partly dissociated words needing to be properly disentangled and formatted. In order to convert the label information into metadata, it is necessary to structure the OCR-extracted text, but so far such work has yet greatly relied on manpower^21^. Further automation of the label data entry task is required to accelerate specimen digitization.

Natural Language Processing (NLP) is a technology aimed at determining and analyzing contextual nuances of language within a document. A major part is automated "information extraction" by which specific text is recognized and presented in a structured form^22^. Named Entity Recognition (NER)^23^ is a system that extracts named entities such as place names, proper nouns, and time from unstructured text. Since the information recorded with specimens in natural history collections can be tagged as named entities, such as scientific names, locality, collector names, and dates, we considered to label the data extracted by OCR with specific named entity extraction codes from which a database would automatically be created.

The idea of applying natural language processing technology to biodiversity science for automatically extracting specific information from various kinds of documents had previously been proposed^24^. A feasibility study has also been conducted to determine whether label data from natural history collections can be extracted semi-automatically from digital images of plant specimens using OCR and natural language processing^25^. Using 250 scanned sample images along with label data manually input by humans (gold standard), the attempt has been made to extract country names, personal names, and place names from texts extracted from the sample images using a system trained by Stanford NER (The Stanford Natural Language Processing Group 2018). That research had tested the feasibility of using OCR and NER to extract and structure sample label information, but a corresponding system was actually not developed at that time.

We here report the successful development of a system that automatically generates a specimen database by first creating a corpus of herbarium specimen labels and then structuring the text data obtained from specimen images using OCR and NER.

## Results

When we searched the text for the parts that matched the scientific name dictionary and the place name dictionary, the number of matching parts was less than half (Table 1). Therefore, we decided to extract the named entity using machine learning. Table 2 shows the results of k-fold cross validation (k = 5, 10) for the three natural language processing libraries, BERT, Albert, and SpaCy. Among them, SpaCy had the highest F-value for both k = 5 and 10, followed by BERT by a very small margin, and Albert having the lowest result. Although the difference between BERT and SpaCy is negligible, we decided to use SpaCy for subsequent application development for versatility, since SpaCy runs on ordinary desktop machines or servers, whereas BERT requires a GPU to run.

**Table 1:** Number of matched cases using OCR text extracted from 10,000 herbarium specimen images.

NER labels	Meaning	Matched cases
en_family_name	Family name of the plants (English)	1880/10,000
'jp_family_name	Family name of the plants (Japanese)	3124/10,000
'en_name	Scientific name	2637/10,000
jp_name	Japanese name	7009/10,000
collect_country	Country	0/10,000
collect_pref	Prefecture	6830/10,000
'collect_city'	Locality	5802/10,000
collect_addr	Street	3954/10,000
collect_date	Date of collection	0/10,000
collect_person	Collector(s)	4381/10,000
collect_number	Collector number	8757/10,000

**Table 2:** Result of K-cross validation (k = 5, 10) applied for the three NLP libraries.

k	Num_entities	Num_predictions	Num_correct	Precision	Recall	f_value
k = 5						
Bert						
Mean	2165.2	2301.4	1662.2	0.722	0.768	0.744
S.D.	35.1	54.4	41.6	0.015	0.014	0.014
Albert						
Mean	2165.2	2292.0	781.8	0.343	0.361	0.351
S.D.	35.1	183.6	42.1	0.030	1.015	0.020
SpaCy						
Mean	2165.2	2089.2	1630.2	0.781	0.753	0.766
S.D.	35.1	91.0	34.1	0.020	0.016	0.006
k = 10						
Bert						
Mean	1082.6	1140.7	842.8	0.739	0.779	0.758
S.D.	35.6	36.2	27.5	0.016	0.017	0.016
Albert						
Mean	1082.6	1263.9	452.5	0.359	0.419	0.386
S.D.	33.6	54.7	29.9	0.033	0.034	0.033
SpaCy						
Mean	1082.6	1050.2	821.1	0.782	0.759	0.770
S.D.	33.6	38.2	20.6	0.016	0.019	0.015

Table 3 shows the results of k-fold cross validation with three types of data (manual corpus data, manual + 10,000 artificial data, and 10,000 artificial data only) given to SpaCy. The best results were obtained when the manual corpus + artificial data were given, followed by the manual corpus data only, and the lowest F-value was obtained when the artificial data only were given. A demonstration of the developed application can be viewed at https://youtu.be/2jt_GMUqrWQ.

**Table 3:** Result of k-cross validation (k = 5, 10) applied for SpaCy with manual corpus, artificial data, and combined data.

k	Num_entities	Num_predictions	Num_correct	Precision	Recall	f_value
With manual corpus						
k = 5						
Mean	2165.2	2089.2	1630.2	0.781	0.753	0.766
S.D.	35.1	91.0	34.1	0.020	0.016	0.006
k = 10						
Mean	1082.6	1050.2	821.1	0.782	0.759	0.770
S.D.	33.6	38.2	20.6	0.016	0.019	0.015
With manual corpus + artificial data (10,000)						
k = 5						
Mean	2165.2	2056.2	1688.8	0.821	0.780	0.800
S.D.	35.1	33.9	22.0	0.005	0.004	0.002
k = 10						
Mean	1082.6	1031.7	843	0.817	0.779	0.798
S.D.	33.5	33.4	19.8	0.013	0.022	0.016
Artificial data only (10,000)						
k = 5						
Mean	2165.2	1365.6	1063.4	0.779	0.491	0.602
S.D.	35.1	16.3	18.6	0.008	0.012	0.011
k = 10						
Mean	1082.6	682.8	531.7	0.779	0.491	0.602
S.D.	33.6	15.5	15.5	0.011	0.015	0.013

Label data entry using the new data entry system resulted in 19–20 labels per hour in average. As they became more familiar with the system, they were able to input even faster.

Interested users who would like to test the system for a limited amount of time can apply to the corresponding author by e-mail to receive a password for database access.

## Discussion

We show that data augmentation indeed improves the results of our model. As seen in Table 3, the F-value was highest when a manual corpus plus artificial data was used, followed by training using a manual corpus alone. Machine learning requires a huge amount of training data, but it is often difficult to prepare such a large amount of data. In such a case the use of artificial data is beneficial^26,27^. In our case, artificial data alone could not substantially improve the learning effect, but data augmentation was able to improve the results of the model. It thus is necessary to manually create a high-quality corpus as well as appropriately generated artificial data to improve the accuracy of the model.

**Figure 1:**
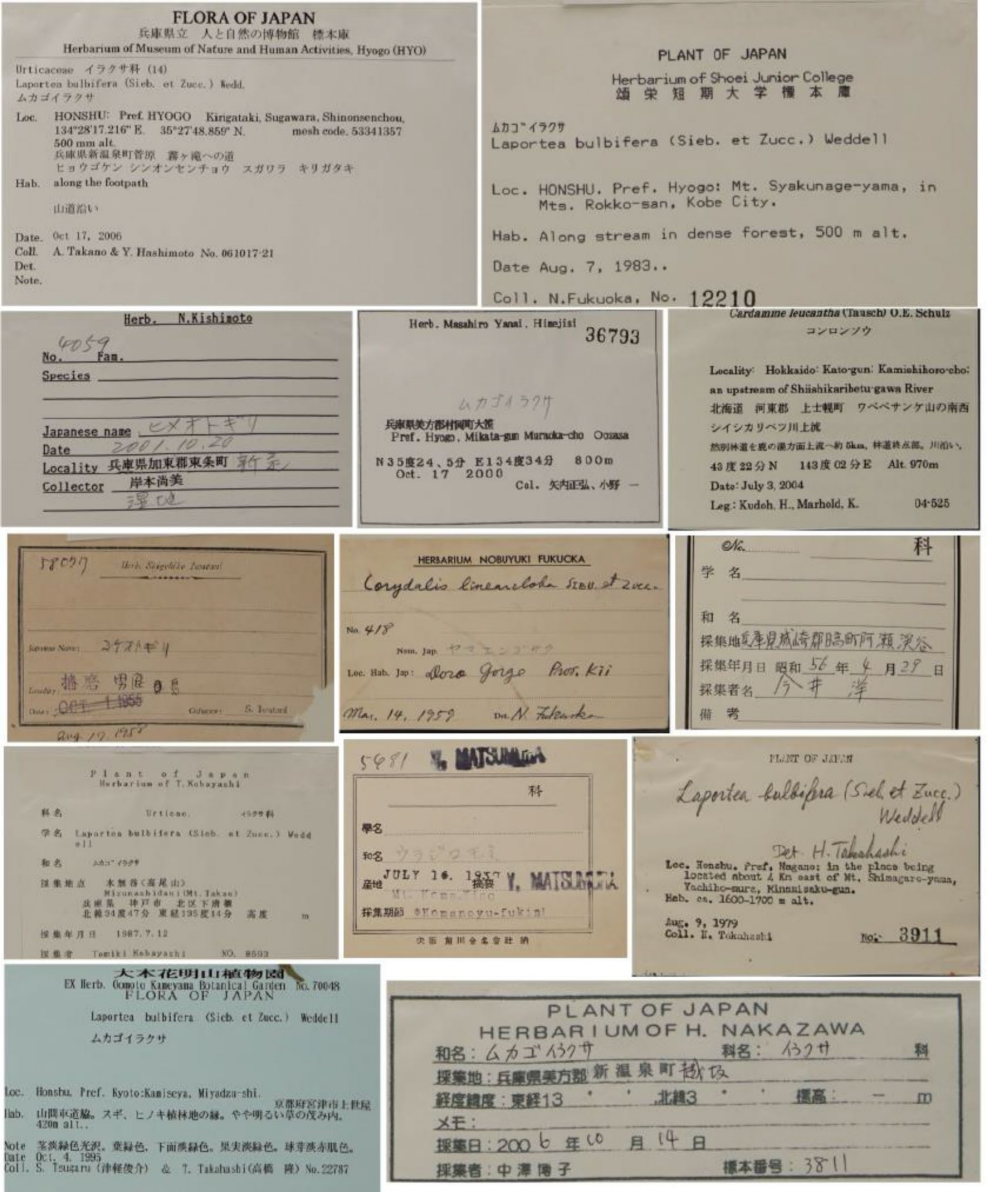
Different kinds of labels on herbarium specimen sheets at HYO.

Application of the new system developed and described here will help to increase the mobilization of herbarium specimens in Japan. Many systems for label data input by OCR have been developed in Europe and the USA^13–16^. However, it has so far been impossible to apply this technology in Japan, the reason being the uniqueness and complexity of Japanese specimen labels. Specimen labels produced by Japanese collectors are written in several languages/scripts, with the locality and collector's name in Japanese or both Japanese and English, and the scientific name of the plant in Latin (Fig. 1). Automated text extraction requires the use of multilingual OCR, and it was necessary to develop a label data extraction system specifically for images of Japanese herbarium specimens. Japan has 178 active herbaria with a total of 15 million specimens^28^. Although Japan has lagged behind other countries in digitizing herbarium specimens^10^, specimen photography equipment developed by Takano et al. 2019 now has been used at the University of Tokyo (TI), Kyoto University (KYO), Osaka Museum of Natural History (OSA), and others, and specimen digitization is now performed in several large herbaria. However, for specimen mobilization, it is essential to generate metadata as well as to take digital images. The development of this automated label data input system can help to promote metadata extraction and further advance the mobilization of herbarium specimens in Japan and beyond.

While the application presented here is specifically designed for Japanese herbarium sheet labels, it is suitable for supporting automated sample label reading of any country or language by applying a suitable and well-trained NLP library along with the corresponding learning dictionaries and data sets, since the code of the application is available in GitHub. Applying this system in other countries may help to promote the digitization of plant specimens all around the world.

## Methods

Images of 20,000 herbarium specimens were prepared at the Museum of Human and Nature Activities, Hyogo (HYO), in conjunction with using the database of scientific names of Japanese vascular plants set up at HYO and the database of Japanese postal codes (https://www.post.japanpost.jp/zipcode/download.html). Labels for named entity recognition were set as shown in Table 4.

**Table 4:** Labels used in the Named Entity Recognition (NER) in this study.

Label	Meaning
en_family_name	Family name of the plants (English)
'jp_family_name	Family name of the plants (Japanese)
'en_name	Scientific name
jp_name	Japanese name
ja_pref*	Prefecture (Japanese)
ja_city*	Locality (Japanese)
ja_addr*	Street (Japanese)
en_pref	Prefecture (English)
en_city	Locality (English)
en_addr	Street (English)
date	Date of collection
person	Collector(s)
number	Collector's number
country	Country
lat*	Latitude
long*	Longitude
alt*	Altitude
memo*	Memo

*Asterisk indicates the labeling using deep learning only.

### Named entity recognition (NER) using dictionary-based text matching

Using 10,000 sample images, we first tried a dictionary-based text matching method. The method extracts from the input text the parts that match the entries in the dictionary as classes of the matching dictionary entries. The label part was detected from the sample image^18^, the text was extracted using Google OCR, and the text information was stored in a column.

### Preparation of NER by machine learning

#### Creation of a corpus

**Figure 2:**
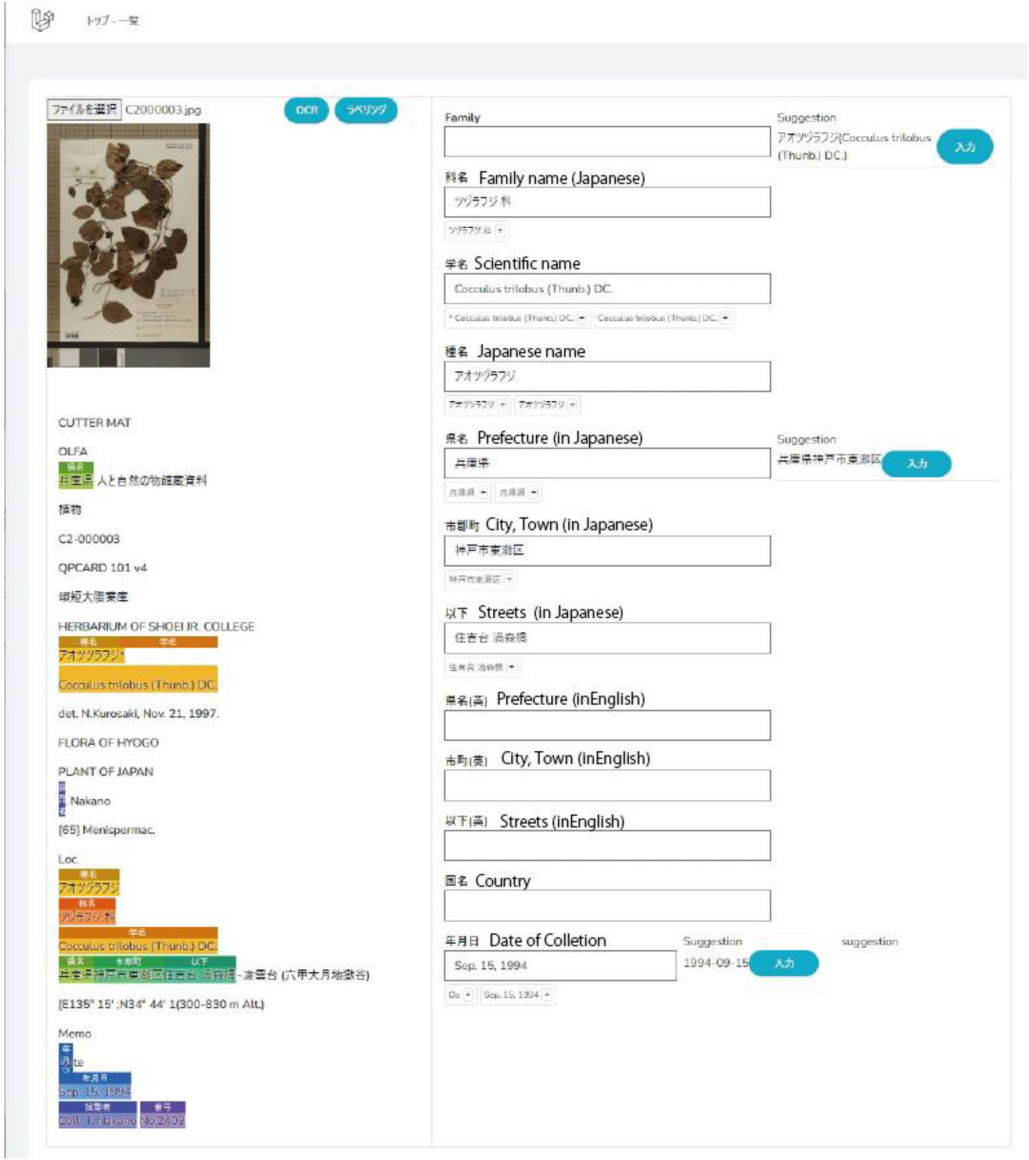
Example of an application for labeling OCR-extracted text label data. The text extracted from the specimen image in the top left-hand corner is displayed in the left-hand column. Labels suggested by the system are displayed in different colors. When labels are modified, changed, etc., in the text in the left-hand column, the changes are reflected in the right-hand column.

In order to perform NER based on classification by machine learning, we manually created a corpus of herbarium specimen labels used for test data and training data manually. As a strategy, we gave top priority to covering the variation of specimen labels found in 20,000 HYO specimen images. Usually, specimen labels are created by the person who collected the plants. The information written on the specimen labels is roughly the same for plant name, collection site, name of the collector, collection date, etc. The format of the labels differs depending on the collector, so they are variable (Fig. 1). Depending on the collector, the collector’s number may be above or to the bottom right of the label, and the collector`s name may also be in the bottom right or bottom left. As the format of the labels varied, we decided to let the machine learning models learn the various label formats in order to achieve accurate data extraction. Collectors tend to continue to create labels in the same format, therefore, we grouped the data for each collector, ranked them randomly, and selected the first to third ranked ones as the target of creating learning data. In order to create an efficient corpus, we created a learning data creation tool (a web application) (Fig. 2). The application uses Laravel, a PHP framework, and runs in graphical user interface (GUI) and on the command line (CLI).The project files are available on GitHub (https://github.com/HajimeKonagai/HitohakuAI-Laravel). Laravel version 10.24.0 environment is required to use the application. Given a set of specimen images and metadata, the application can prepare teaching data to cover variations in label description style by grouping specimen images by collector and randomly selecting one or more images from each group. The image data after detection from each collector group can be found at the following URL (https://data.hitohaku-ai.jp/images.zip). Since the extraction was done randomly by each collector, the results obtained in this study will not be consistent with those obtained in the replication. In addition, specimens of endangered species were excluded to avoid disclosing information on the collection sites). The specimen images selected above were subjected to OCR. Based on our experience of comparing various OCRs in the past^17^, we chose OCR by Google Cloud Vision. The text resulting from OCR was manually annotated based on the tags in Table 4. The annotated data were used to generate training/teaching data in json format. From the number of names of the collectors and the number of herbaria exchanging specimens with HYO, it was estimated that there were about 1,000 kinds of label formats among the 20,000 specimens. Therefore, we annotated the named entities using an annotation tool, and created 893 training data.

#### Data augmentation

Since the manual annotation method is labor-intensive, 10,000 artificial data were generated as a supplement. Data augmentation was carried out for each item. Family names (Japanese and English), scientific names, and Japanese names were randomly extracted from the botanical name database of HYO. Each data item of place names (Prefecture, City, etc.) was randomly extracted from the national address database of Japan Post and the 20,000 input data of HYO with a probability of half and half. For the collection date, a random date was generated, and various formats were set as follows (e.g., March. 13.1988, 10. Oct. 2003, 16. March, 1997, May. 16.1972, 1979/4/1, 1. Aug. 2005, 24. May, 2013, 12. January, 1975, Mar. 28. 2009, December 30, 1988). For the collector name, a random name was extracted from the data of HYO. For the collector number, a random number was generated between 1 and 999,999. For latitude and longitude, random values were generated in various formats as follows (e.g., − 15° 03′ 1.6, − 70° 43', 24′ 03′ 45, − 152 48, 23° 33′ 09, 34° 44', 67° 48, − 131′ 21, − 86′ 35′ 26, 70′ 53′ 28). Altitude is usually described: either in meters or as a range from xx m to xx m (ex. in the right center of Fig. 1, Takahashi 3911). Therefore, a number was generated randomly between 0 and 8000, and a single notation or a range of xx to xx m was generated for the altitude. Any additional memo information on the labels was not considered as it is too diverse.

#### Evaluation of three NLP libraries

We prepared an application for machine learning (https://github.com/HajimeKonagai/HitohakuAI-Python), where we trained three Natural Language Processing (NLP) libraries and used k-fold cross validation to measure the generalization ability of the algorithm to determine which one to adopt. After loading the pre-trained Japanese data into three NLP libraries (SpaCy, BERT and Albert), the three algorithms were evaluated by using k-fold cross validation. The model is constructed by dividing the data into k groups, with each group as test data and the remaining data as training data, and measuring the accuracy of the model. The k-fold cross validation was set to k = 5 and 10. The pre-trained data and the size of the models at the time of parameter storage were as follows: 1. SpaCy: 'ja-ginza' (5.55 MB), 2. BERT: 'cl-tohoku/bert-base-japanese-whole-word-masking' (419 MB), 3. Albert (lightweight model for BERT): 'ken11/albert-base-japanese-v1-with-japanese-tokenizer' (43.4 MB). The system environment was as follows: HP Z4 G4 Workstation, Processor: Intel (R) Xeon (R) W-2223 CPU @ 3.60 GHz, Implemented RAM 32.0 GB (31.7 GB available), System Type: 64-bit Operating System, × 64-based processor, Edition: Windows 10 Pro for Workstations, Storage: NVMe, GPU NVIDIA RTX A4500.

After having decided on the NLP library, again we performed k-fold cross validation with three types of data (1. manually generated corpus only, 2. artificially generated corpus of 10,000 cases only, and 3. manual + artificially generated corpus of 10,000 cases), with k = 5 and 10.

### Evaluation of the automated data entry application developed in this study

The speed of label data input was examined when using the label data entry system developed here. We asked workers who normally input label data using the OCR-based label data input system^17^ to input data using the newly developed system, and measured how much data could be input per hour.

## Data Availability

The following data sets can be accessed at the corresponding URLs: 1. Dictionary of plant names (945.8 KB) https://data.hitohaku-ai.jp/plant-dict.csv. 2. Dictionary of address (10.9 MB) https://data.hitohaku-ai.jp/address-dict.csv. 3. Manual corpus for teaching data (318.7 KB) (specimens of endangered species were excluded) https://data.hitohaku-ai.jp/annotation.zip. 4. Artificial data (28 MB) (specimens of endangered species excluded) https://data.hitohaku-ai.jp/artificial.zip. 5. Specimen images (2.88 GB) (those of endangered species were excluded) https://data.hitohaku-ai.jp/images.zip. Specimen information used in this study (HYO C2-000001–C2-020000) is openly available in the Global Biodiversity Information Facility (GBIF. https://www.gbif.org/), with the exception of that for endangered species.
